# An Experimental Study of Possible Post-War Ferronickel Slag Waste Disposal in Szklary (Lower Silesian, Poland) as Partial Aggregate Substitute in Concrete: Characterization of Physical, Mechanical, and Thermal Properties

**DOI:** 10.3390/ma14102552

**Published:** 2021-05-14

**Authors:** Marcin Małek, Mateusz Jackowski, Waldemar Łasica, Kamil Dydek, Anna Boczkowska

**Affiliations:** 1 Faculty of Civil Engineering and Geodesy, Military University of Technology, ul. Gen. Sylwestra Kaliskiego 2, 00-908 Warsaw, Poland; marcin.malek@wat.edu.pl (M.M.); waldemar.lasica@wat.edu.pl (W.Ł.); 2 Faculty of Materials Science and Engineering, Warsaw University of Technology, ul. Wołoska 141, 02-507 Warsaw, Poland; kamil.dydek@pw.edu.pl (K.D.); anna.boczkowska@pw.edu.pl (A.B.)

**Keywords:** ferronickel slag waste, by-products, waste disposal, recycling, eco-efficient concrete mixture, concrete modifications

## Abstract

Aggregates derived from waste, due to the growing awareness of global warming, are more and more often used in the concrete production process. This way, their disposal not only reduces the pollution of the Earth but also lowers the consumption of natural aggregates, which are limited. One of the new “eco” aggregates may be a ferronickel slag waste (FNSW), which was generated in post-war metallurgical processes and stored in Szklary (Lower Silesian, Poland). In order to determine the possibility of using ferronickel slag waste aggregate (FNSWA) in the concrete production process, new concrete mixtures were designed and tested. Physical properties (cone slump, air content, pH, and density), mechanical properties (compressive strength, flexural strength, and tensile strength), and thermal properties (thermal conductivity) were assessed for all new laboratory recipes. Moreover, the modulus of elasticity and Poisson’s ratio were determined. This study includes five different contents of FNSWA in the amount of 5%, 10%, 15%, 20%, and 25% of the mass of natural aggregate as its partial substitute. The final results were compared to the base sample (BS) containing 100% natural aggregate, which was granite.

## 1. Introduction

The war period was particularly severe for Poland. A significant number of the country’s population died, defending their homeland, some lands were taken away, and the losses in the infrastructure were difficult to accurately quantify. It is estimated that the war cost that one resident had to pay was around USD 626 [1]. Despite the initial difficulties however, the reconstruction of the country has begun, thus implementing recovery plans. One of them, the Six-Year Plan, assumed the development of the metallurgical industry due to the large amount of natural resources and minerals present in Poland. The priority of the plan was to increase investment and the policy of intensive induction of the country on the soviet model. According to the Six-Year Plan, the industrial production alone was to increase by 85–95% [2,3,4], which could not have had a good environmental impact.

The post-war development of the polish industry has left its mark on the environment by leaving soil and groundwater contamination, and a large amount of waste that remains to this day [5,6]. This phenomena is especially visible in Silesian Districts of Southern and South-Western Poland where coal-waste dumps are stored. The enormous amounts of material from coal mining and coal production, due to the intense coal exploitation that has begun in the 19th century, is accumulated on dumps [7]. These materials are exposed to processes of self-heating, water-washing, and biodegradation, which can lead to significant environmental problems around coal-waste dumps, such as high concentrations of toxic compounds in soil or acidic gases and chemicals in the air [8]. Therefore, not only the environment but also human and animal life may be in danger. However, coal-waste is not the only type of waste left after the post-war development of the polish industry. Another big problem lies in South-Western Poland in Lower Silesian voivodeship. A small village, Szklary, within Ząbkowice Śląskie County, is a place where up until 1982 the only nickel ore mine in Poland was located. It has been officially closed due to the unprofitability of further exploitation but huge amounts of ferronickel slag waste are accumulated there to this day [9,10,11]. As it is stored in dumps, similar processes to coal-waste dumps occur. This work is a proposal of ferronickel slag waste utilization in concrete mixtures to prevent further contamination of groundwater, soil, and air spreading across Ząbkowice Śląskie County, especially Szklary.

Waste and by-products from other industry branches can be re-used by the construction industry as a component of concrete and mortar [12,13,14,15]. According to current studies, they can be used as a replacement of natural aggregate [16] and cement [17], as dispersed reinforcement [18,19,20,21] to lower surface stress and prevent microcracks or as a filler to increase thermal properties of final composite [22]. Ferronickel slag waste has a potential to substitute natural aggregate as it is a solid discharged from the smelting process of metal nickel or nickel-iron alloy. What is worth mentioning is that it has a high density and durability. Such properties are obtained in the heating and calcining process of nickel, where it is bonded with iron oxide. Moreover, this fusion allows high resistance to corrosion, heat shock, and fatigue, as well as the ferromagnetic effect of the final material [23].

The possibility of ferronickel slag waste utilization in concrete has been studied during recent years by Saha et al. [24,25,26,27]. As they reported, a 50% replacement of natural sand with FNSWA increases compressive strength from 38 to 57 MPa in concrete [26]. What was unexpected, other mechanical properties also showed higher values compared to plain concrete. Saha et al. noted about 7% and 13% increase in split tensile strength and flexural strength, respectively. Similar trends were observed by Nguyen et al. [28] as they reported an increase in concrete performance containing ferronickel sand as a fine aggregate. They pointed out, that both, mechanical and durability properties increased, due to the better interfacial transition zone between ferronickel aggregate and cement paste. Nuruzzaman et al. [29] also tested ferronickel slag waste as an aggregate replacement. With the density of 2920 kg/m^3^ and substitution in range from 20% to 60% they achieved an increase in concrete mixture workability and a decrease in water absorption of hardened samples. Furthermore, concretes containing 20%, 40%, and 60% showed an increase by 1%, 34%, and 31% compressive strength compared to the base mix (37 MPa), and an increase by about 2%, 15%, and 8% split tensile strength compared to the base mix (4.3 MPa), respectively. The same trend after replacing coarse aggregate with ferronickel was reported by Sun et al. [30] and Qi et al. [31]. However, not only mechanical and psychical properties changes have been reported after FNSWA incorporation in concrete mixture. As presented by Saha [32], ferronickel slag can lower the thermal conductivity, as well. A concrete designed by them, with 100% FNSWA, showed 1.16 W/mK thermal conductivity, which was about two times lower compared to the base mix with only natural aggregate (2.34 W/mK).

This study aims to contribute to this growing area of research by exploring the potential usage of FNSWA mined in Szklary (Lower Silesia, Poland) as a replacement of natural aggregate in concrete. The problem of FNSW in Lower Silesia in Poland is very serious, so far, however, no possible method of utilization of this waste has been efficiently introduced to the Polish industry. To do so, psychical, mechanical, and thermal properties on new concretes were tested. Findings presented in this study make an important contribution to the effective utilization of ferronickel slag waste accumulated in Szklary (Lower Silesia, Poland) and contribute to the environment harmed by ferronickel slag waste stored there. As it has a different mineral composition proportion compared to the ones presented in current studies it may also show different results of concrete properties, especially its mechanical strength. Moreover, such a replacement is consistent with the principle of sustainable development and the circular economy.

## 2. Materials

### 2.1. Specimen Preparation

**Figure 1:**
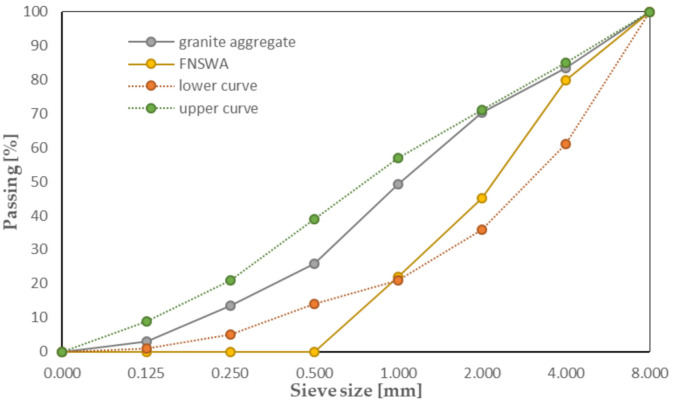
Gradation of crushed granite aggregate and FNSWA.

Table 1 shows chemical composition and strength parameters of the Portland cement (CEM I 42.5 R [33]) used for each concrete mixture, which were determined according to EN 196-6:2019-01 [34] and PN EN 196-1:2016-07 [35]. Moreover, all of the recipes were based on tap water (chloride content 28 mg/l) and natural granite aggregate, used as a filler, with a fraction up to 8.0 mm. Its grain size index (C_U_ = 7.2 and C_C_ = 1.1) suggests that aggregate is well compacted [36] and, as presented in Figure 1, fits between upper and lower curves determined in accordance with the EN 12620+A1:2010 [37] standard.

In order to increase the workability of all mixtures, but at the same time maintain the water-cement ratio *w*/*c* at 0.44, the chemical admixture was used. It was a low-alkaline liquid, free of chlorine, and based on an aqueous solution of modified polycarboxylic ethers (melamine and silanes/siloxanes), which helped reduce the accumulated water on the surface of concrete after compaction, as well [39]. The chemical composition of the chemical admixture is given in Table 2.

### 2.2. Ferronickel Slag Waste

**Figure 2:**
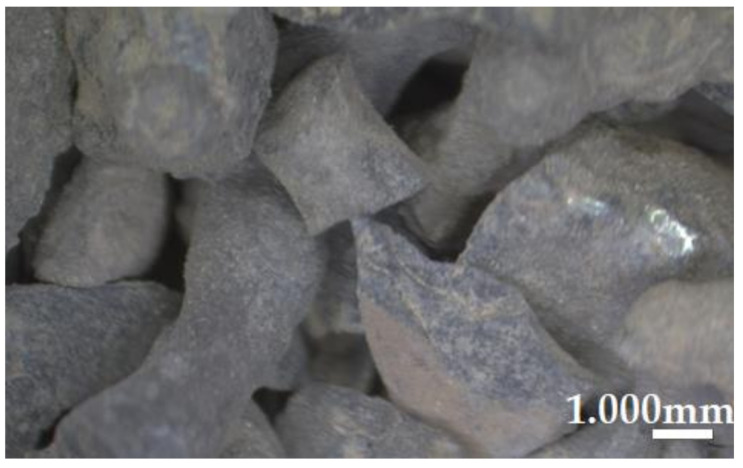
Ferronickel slag waste aggregate.

As a partial substitute to natural granite aggregate, ferronickel slag waste was used (Figure 2). FNSW was mined from the ferronickel waste dump stored in Szklary, within Ząbkowice Śląskie County (Lower Silesia, Poland) and transported to the laboratory.

Table 3 presents the chemical composition of FNSW used as an aggregate substitute, the density of which is about 2750 kg/m^3^. Compared to cement, its chemical composition is similar due to the fact that it contains a significant part of the compounds present in the binder. The FNSWA sieve curve is given in Figure 1.

### 2.3. Mix Composition

Six different types of mortar mixtures were produced, see Table 4. The FNSWA content, which was added rather than the same amount of natural aggregate, was 5%, 10%, 15%, 20%, and 25% of granite mass for samples marked in the article as 5FNSW, 10FNSW, 15FNSW, 20FNSW, and 25FNSW, respectively. The sample with 100% of natural granite aggregate was marked as BM and used to compare test results.

### 2.4. Mix Production

After mixing dry components for 1 min, the liquid components were added to the concrete mixture, and blended together for 4 min. Next, fresh concrete was poured into molds and compacted on a vibrating table. All of the samples were manufactured in 21 °C temperature and 50% humidity inside a laboratory and stored in water according to EN 12390-2:2019-07 [40]. After 28 days, the samples were tested for each property.

From each mix, the following samples were produced: Ten cubes with dimensions of 150 × 150 × 150 mm, five cuboids with dimensions of 40 × 40 × 160 mm, five rollers with a base diameter of 150 mm and a height of 300 mm, and one tile with dimensions of 30 × 30 × 6 mm. In total, 60 cubic samples, 30 cuboid samples, 30 cylinders, and six tiles were made to conduct this study.

## 3. Methodology

### 3.1. Fresh Concrete Tests

The slump cone test was carried out according to EN 12350-2:2019-07 [41], when the air content and pH value of the mix were determined according to EN 12350-7:2019-08 [42] and PN-B-01810:1986 [43], respectively. Five samples for each mixture were investigated. These tests were performed in a listed order and right after the mixing process was done. The presented values for the conducted tests are the average values of five samples made in pursuance of each concrete recipe.

### 3.2. Hardened Concrete Tests

The density of concrete samples, was determined according to EN 12390-7:2019-08 [44] on 150 × 150 × 150 cubes. Furthermore, mechanical properties were assessed, such as compressive strength, flexural strength, and tensile strength. To do so, a Zwick machine (Zwick, Ulm, Germany) with a force range of 0–5000 kN and maximum stress increase of 0.5 MPa/s was used. The compressive strength was measured according to EN 12390-3:2019-07 [45] on 150 × 150 × 150 mm samples, which were placed in the Zwick machine. Next, the compressive strength of each test was determined by dividing the maximum sample load value by the sample cross-sectional area. For the flexural strength test, in a three-point bending set-up, 40 × 40 × 160 mm concrete beams were prepared according to EN 12390-5:2019-08 [46]. The Zwick machine used for the test enabled loading with a static force and keeping it in a vertical configuration at a constant assumed level thanks to the supports that allowed only the horizontal movement (the distance between supports was 100 mm). The last mechanical property, the split tensile strength, was tested on cylinders of 150 mm diameter and 300 mm height according to EN 12390-6:2011 [47]. In order to perform the test, the sample was placed in the Zwick machine on sliding supports, immobilized in the guides of the testing machine. The distance between the supports was greater than 10 diameters of the sample and the pressure head and supports were radially rounded at the point of contact with the sample. The cylinders of 150 mm diameter and 300 mm were also used to define the Modulus of elasticity and Poisson’s ratio according to the EN 12390-13:2014-02 [48] standard. Every sample was loaded and the load was removed in the lower and upper stress range to determine these material properties of each hardened concrete mixture. The modulus of elasticity and Poisson’s ratio were determined thanks to linear displacements and measuring base lengths noted with Epsilon extensometers (Epsilon, Jackson, WY, USA). The scope of the research also included thermal conductivity. The temperature responses to the material heat flow pulses were the basis for all measurements done with the ISOMET2114 analyzer (Applied Precision Ltd., Bratislava, Slovakia). The analyzer probe, containing the resistor heater through which the heat flow was induced, had a direct contact with the tested sample. A probe with a diameter of 60 mm and the tested material in the form of cubes (150 × 150 × 150 mm) from each concrete mix were used.

**Figure 3:**
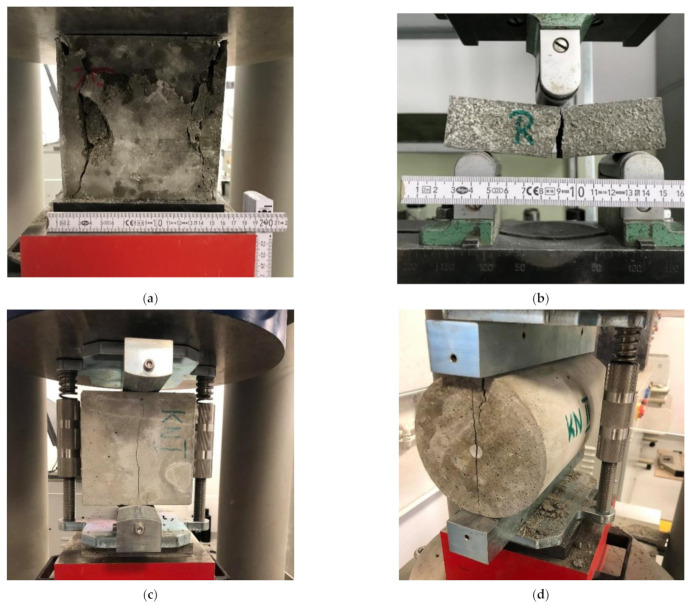

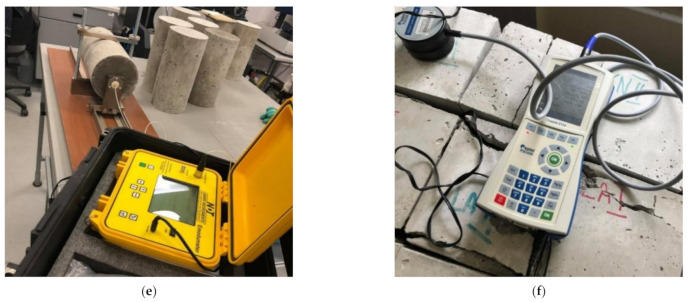
Photographic documentation of tests carried out: (**a**) Compressive strength test, (**b**) flexural strength test, (**c**) split tensile strength test—cubes, (**d**) split tensile strength test—cylinder, (**e**) elastic modulus test, (**f**) thermal conductivity test.

All of the specified above tests were carried out after 28 days of the curing process. Each of them was performed on five samples of every mix and the values presented are the average values (Figure 3).

## 4. Results and Discussion

### 4.1. The Slump Cone Test

Table 5 shows the slump cone test results of all concrete recipes. According to EN 12350-2:2019-07 [41], they could be classified as a S1 class. Analyzing the obtained results, it can be seen that the modification with ferronickel slag did not affect the cone slag at any percentage share of the natural aggregate substitute, and thus the fresh concrete consistency. All of the presented values of the slump cone fall test are in the error range. The slump cone test was also conducted by Saha et al. in their studies [24,26]. They reported however a much higher fall as the values fluctuated in the range of 200 to 230 mm and in the range of 120 to 150 mm for mortars and concretes with ferronickel slag addition, respectively. This difference in the results may be due to the higher water/cement ratio used by Saha and the smaller size of ferronickel slag waste compared to our study.

### 4.2. Air Content

The air content presented in Table 5 shows that the peak value of this property was reported for the 25FNSW sample (modified with 25% of ferronickel slag waste). It showed an increment of 1.5% compared to BM. Previous substitutions of granite aggregate also indicated a higher air content in concrete mixtures compared to BM. This phenomena may be a result of the higher grain size of FNSWA compared to the granite aggregate used in the concrete manufacturing process. It can also indicate more air voids both in fresh and hardened concrete.

### 4.3. The pH Test

As presented in Table 5, the pH of all concrete mixtures exhibits the alkaline reaction. The highest value of pH was reported for samples with 25% ferronickel slag waste aggregate (12.72). Compared to the base mix values (12.61) an increase of about 1% can be noted. Such a small change may indicate that FNSWA did not affect the pH of the concrete mixture significantly.

### 4.4. Density

**Figure 4:**
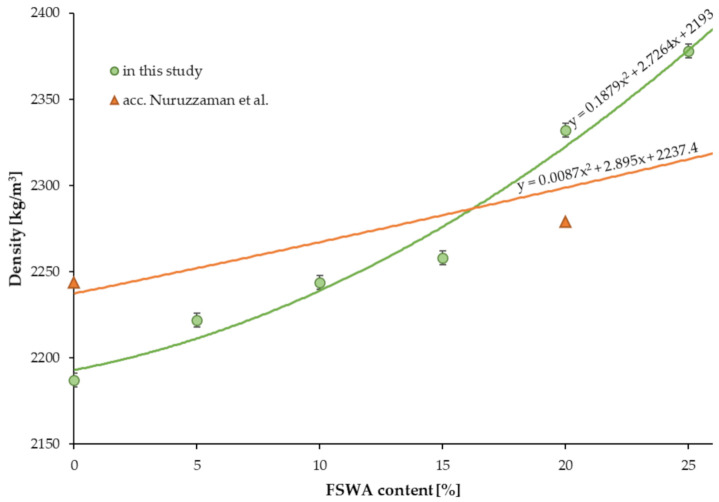
The impact of FNSWA on concrete density.

The density of concrete samples is presented in Table 6. The substitution equal to 5%, 10%, 15%, 20%, and 25% of natural aggregate done with FNSWA, resulted in a density increase by 1.6%, 2.6%, 3.2%, 6.6%, and 8.7% compared to the BM, respectively. All of the obtained values were in the range between 2187 ± 2 kg/m^3^ (BM) and 2378 ± 3 kg/m^3^ (25FNSW), and, after analyzing them, an overall trend can be noted as: The higher the FNSWA amount, the higher the density of concrete, see Figure 4. This may be a result of the higher density of FNSWA compared to the natural granite aggregate. Similar to this study, Nuruzzaman et al. [29] also reported an increase in density of concrete containing FNSWA. As they reported, the density increased by 1.58%, 3.15%, and 4.73% due to the use of 20%, 40%, and 60% FNSWA. On the other hand, the opposite trend was reported by Bouasria et al. [49]. They, however, replaced not the aggregate but the cement with ferronickel slag, and noted a decrease in concrete density from 2390 to 2330 kg/m^3^ after a 10% substitution. Further modifications also showed decreased values—2310 and 2300 kg/m^3^ for 15% and 30% replacement of cement, respectively. This may be connected to a higher density of cement powder compared to ferronickel slag.

### 4.5. Compressive Strength

**Figure 5:**
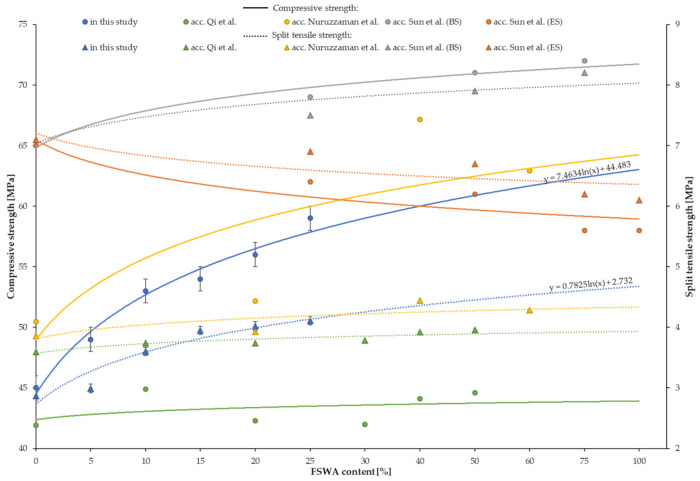
The impact of FNSWA on compressive strength and split tensile strength.

The results of compressive strength for samples with different contents of FNSWA are presented in Table 6 and in Figure 5. The results were compared to the base mix (without FNSWA), which showed a compressive strength of 45 ± 1 MPa. All of the substitution of natural aggregate, equal to 5%, 10%, 15%, 20%, and 25%, indicated an increase in compressive strength by 8.8%, 17.8%, 20.0%, 24.4%, and 31.1%, respectively. For this case, the highest value of compressive strength (59 ± 1 MPa) was obtained for samples with the highest amount of FNSWA (400 kg/m^3^). The same phenomena was reported by Qi et al. [31] as they noted the peak values of compressive strength for samples modified with 50% of FNSWA (44.6 MPa). Another study in line with this trend was published by Nuruzzaman et al. [29]. The authors tested the FNSW concrete with ferronickel slag grading up to 9.50 mm and density of 2920 kg/m^3^ with its addition equal to 20%, 40%, and 60%. They reported similar values of compressive strength (52.15 MPa) for samples containing 20% of ferronickel slag compared to 20FNSW samples (56 ± 1 MPa). Moreover, Sun et al. [30] used two types of ferronickel slag for concrete manufacturing—blast furnace slag (BS) and electric furnace slag (ES). They showed that BS indicates an increase in compressive strength by about 6%, 9%, and 11% for samples with 25%, 50%, and 75% of ferronickel used as an aggregate. In the opposition to this were however results of ES concretes, as they presented a decrease in compressive strength with an increase in ferronickel slag content. The opposite trend noted for samples containing ES may be due to the type of ferronickel used, which showed a smooth surface. In turn, it can affect the interactions between components and lower the results of mechanical tests. The lowest compressive strength was reported for samples with 75% and 100% electric furnace slag (58 MPa) and compared to the reference sample (65 MPa), it was a decrease by about 11%.

### 4.6. Split Tensile Strength

**Figure 6:**
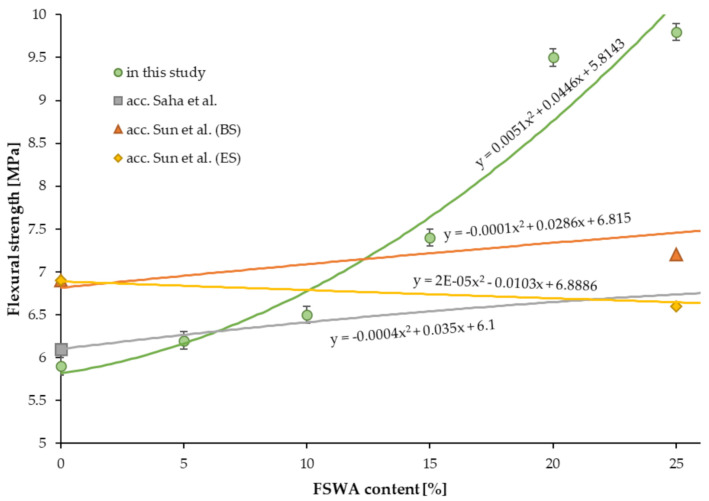
The impact of FNSWA on flexural strength.

Table 6 shows the split tensile strength test results of concrete with a natural aggregate replacement done with FNSWA. After substitution of granite aggregate with 5%, 10%, 15%, 20%, and 25% of FNSWA, the linear increase in split tensile strength was reported, see Figure 6. Compared to BM, the samples modified with FNSWA showed higher values by around 4%, 25%, 38%, 40%, and 43% values, respectively for 5FNSW, 10FNSW, 15FNSW, 20FNSW, and 25FNSW samples. As observed, the difference in split tensile strength for the reference sample and the sample with the lowest amount of FNSWA (80 kg/m^3^) was not significant. However, a further increase in FNSWA used in concrete resulted in a visible increase in the split tensile strength, what also was reported by Nuruzzaman et al. [29]. Their FNSW concrete showed a 2%, 15%, and 11% increase in split tensile strength for 20%, 40%, and 60% ferronickel used as an aggregate. Furthermore, Qi et al. [31] tested FNSWA as a substitute to natural aggregate. They used five different contents of ferronickel equal to 10%, 20%, 30%, 40%, and 50%. As presented in their study, the highest split tensile strength (3.96 MPa) showed samples with 50% of FNSWA, which was about 10% greater than plain concrete. An increasing trend was also reported for BS concretes tested by Sun et al. [30], however their ES concrete showed an opposite phenomena. This may be a result of ES particles that contain a number of smooth glass surfaces, which can seriously weaken the cohesion between cement paste and aggregates, and mechanical properties of hardened concrete.

### 4.7. Flexural Strength

Table 6 presents the results of the flexural strength test of concrete samples with FNSWA. For the 5%, 10%, 15%, 20%, and 25% substitution of natural granite aggregate, the obtained flexural strength was about average 5%, 10%, 25%, 61%, and 66% higher than for the plain concrete (5.9 ± 0.1 MPa), respectively. The flexural strength is directly proportional to the FNSWA amount used in the concrete mixture, which is shown by a linear increase in values. Furthermore, the determined *R^2^* coefficient equal to 0.9, means a good fit. This trend was also reported by Saha et al. [24], as well. They showed however, that a peak in flexural strength is achievable for 50% FNSWA used in concrete and a further increase in the amount of natural aggregate substitute decreases this property. Their study complies with the Sun et al. research [30] as they reported after 28 days a constant value of flexural strength for samples modified with 50% and 75% of ferronickel slag (8.2 MPa). What is worth mentioning is that Sun et al. who used two types of the ferronickel slag described above reported an increase for BS concretes, when the electric furnace slag showed a decrease in flexural strength by about 4%, 6%, 10%, and 12% for 25%, 50%, 75%, and 100% natural aggregate replacement, respectively.

### 4.8. Modulus of Elasticity and Poisson’s Coefficient

The modulus of elasticity was slightly influenced by the aggregate substitution done with FNSWA. Results given in Table 6, range from 31.5 ± 0.4 to 33.6 ± 0.3 GPa. The highest values of modulus of elasticity were reported for 25FNSW samples, which compared to BM showed a 6.7% increase in value. This trend was reported for the rest of the samples as well, as the noted values were higher than the plain concrete (0.3%, 1.0%, 1.9%, and 2.9% increase for 5FNSW, 10FNSW, 15FNSW, and 20FNSW samples, respectively). An increasing trend, after replacing 50% of natural sand aggregate with FNSWA, was reported by Sakoi et al. [50] and Saha and Sarker [26], as well. However, their increase was about 14% and 12%, respectively, which is almost two times higher compared to this study. Such a difference was caused by the greater FNSWA amount used by other researchers, which could result in an increase of modulus of elasticity as it is related with the density of concrete components.

On the other hand, the results of the Poisson’s ratio, presented in Table 6, indicate no influence of FNSWA to this concrete property, as they are in the measurement error range. Therefore, it can be said that the substitution of natural granite aggregate done with FNSWA in the range from 5% to 25% did not affect the Poisson’s ratio. A similar conclusion was made by Qi et al. [31] as they stated that Poisson’s ratio of concrete containing ferronickel slag is consistent with that of conventional concrete.

### 4.9. Thermal Conductivity

**Figure 7:**
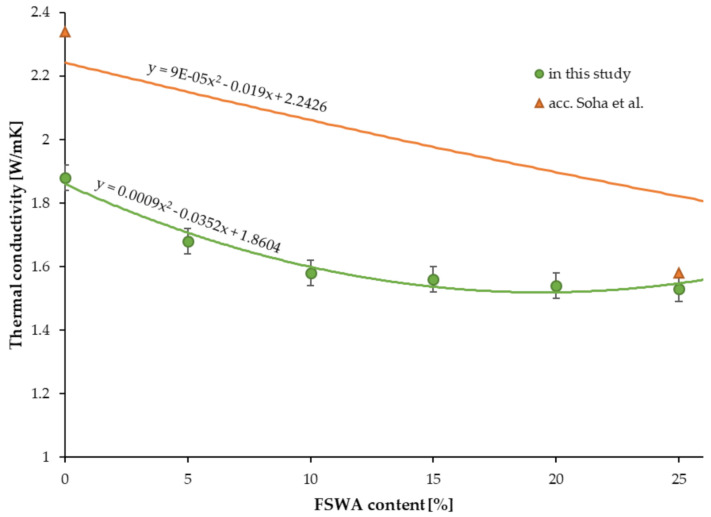
The impact of FNSWA on thermal conductivity.

The obtained results of the thermal conductivity test ranged from 1.53 to 1.88 W/mK (Table 6). The concrete modified with 25% FNSWA showed the lowest values of thermal conductivity, which were 18.6% less than the plain concrete. Moreover, a linear decreasing trend can be reported as thermal conductivity drops with an increase in the amount of the aggregate substitute. The same phenomena was reported by Saha et al. [32]. Their plain concrete reduced its thermal conductivity from 2.34 to 1.58 W/mK, 1.65 W/mK, 1.36 W/mK, and 1.16 W/mK, with 25%, 50%, 75%, and 100% FNSWA replacement, respectively. As presented in Figure 7, the thermal conductivity values reported for concretes with 25% ferronickel slag waste in this study and by Saha et al. are almost equal.

## 5. Conclusions

The aim of this study was to evaluate the possibility of using the ferronickel slag waste aggregate stored in dumps in Szklary (Lower Silesian, Poland) as an aggregate substitute in concrete production. To do so, tests on new laboratory concrete mixtures were carried out, from which final key conclusions can be made:

- The highest air content was reported for 25FNSW samples (3.6%);
- The density of hardened concretes were proportional to the amount of FNSWA used. The density increased with the increasing FNSWA and the highest density (2378 kg/m^3^) was found for concrete with 25% FNSWA and the lowest density (2187 kg/m^3^) for plain concrete;
- The substitution of 80, 160, 240, 320, and 400 kg/m^3^ natural granite aggregate with FNSWA caused an increase in the compressive strength by about 1.09, 1.18, 1.20, 1.24, and 1.31 times and in flexural strength by about 1.05, 1.10, 1.25, 1.61, and 1.66 times, respectively compared to plain concrete, while the obtained split tensile strength was increased compared to BM by 4.0%, 25%, 38%, 40%, and 43%, respectively
- A slight influence of FNSWA on the modulus of elasticity was noted as reported values showed an increase in the range of 0.3% up to 6.7%, depending on the amount of substitute used;
- All of the concretes with FNSWA showed lower thermal conductivity values compare to BM. The highest decrease was reported for 25FNSW samples (18.6%);
- The substitution of natural aggregate done with FNSWA did not affect consistency, the pH value, and Poisson’s ratio of concrete.

This research presents a solution to air, soil, and groundwater pollution caused by the remains of post-war mining activities in Szklary (Lower Silesia, Poland). The set goal of this case study, which was to determine the physical, mechanical, and thermal properties of concretes containing FSWA, was successfully achieved. All of the new concretes showed increased strength parameters, crucial in building materials, which can indicate a great application capacity in the current construction industry.

## Figures and Tables

**Table 1:** Chemical composition and strength parameters of the cement [38].

Compositions	SiO_2_	Al_2_O_3_	Fe_2_O_3_	CaO	MgO	SO_3_	Na_2_O	K_2_O	Cl
Unit (vol%)	19.5	4.9	2.9	63.3	1.3	2.8	0.1	0.9	0.05
Specific surface area (m^2^/kg)	376.3								
Initial setting time (min)	227								
Compressive strength after 2 days (MPa)	28.4								
Compressive strength after 28 days (MPa)	60.8								

**Table 2:** Chemical composition of the admixture.

Compositions	O	Na	Si	K
Unit (vol%)	77.7	14.9	4.8	2.6

**Table 3:** Chemical composition of FNSW.

Compositions	SiO_2_	Fe_2_O_3_	Al_2_O_3_	CaO	MgO
Unit (vol%)	49.8	24.4	11.9	9.6	4.3

**Table 4:** Mix proportions (1 m^3^)**.**

Mix Symbol	Cement [kg]	Water [kg]	Chemical Admixture [kg]	Granite Aggregate [kg]	FNSWA [kg]
BM	468	207	4.7	1600	0
5FNSW				1520	80
10FNSW				1440	160
15FNSW				1360	240
20FNSW				1280	320
25FNSW				1200	400

**Table 5:** Fresh concrete test results.

Mix Symbol	Slump Cone [mm]	Consistency Class [41]	Air Content [%]	pH [-]
BM	2 ± 1	S1	2.1 ± 0.1	12.61 ± 0.03
5FNSW	2 ± 1	S1	2.2 ± 0.1	12.65 ± 0.03
10FNSW	2 ± 1	S1	2.4 ± 0.1	12.68 ± 0.04
15FNSW	2 ± 1	S1	2.7 ± 0.1	12.69 ± 0.03
20FNSW	2 ± 1	S1	3.2 ± 0.1	12.71 ± 0.03
25FNSW	1 ± 1	S1	3.6 ± 0.1	12.72 ± 0.04

**Table 6:** Hardened concrete test results.

Mix Symbol	Density [kg/m^3^]	Compressive Strength [MPa]	Split Tensile Strength [MPa]	Flexural Strength [MPa]	Modulus of Elasticity [GPa]	Poisson Coefficient [GPa]	Thermal Conductivity [W/mK]
BM	2187 ± 2	45 ± 1	2.87 ± 0.03	5.9 ± 0.1	31.5 ± 0.4	0.123 ± 0.03	1.88 ± 0.04
5FNSW	2222 ± 2	49 ± 1	2.99 ± 0.05	6.2 ± 0.1	31.6 ± 0.3	0.124 ± 0.03	1.68 ± 0.04
10FNSW	2244 ± 3	53 ± 1	3.60 ± 0.03	6.5 ± 0.1	31.8 ± 0.3	0.129 ± 0.04	1.58 ± 0.04
15FNSW	2258 ± 2	54 ± 1	3.95 ± 0.03	7.4 ± 0.1	32.1 ± 0.4	0.123 ± 0.03	1.56 ± 0.03
20FNSW	2332 ± 2	56 ± 1	4.02 ± 0.03	9.5 ± 0.2	32.4 ± 0.3	0.124 ± 0.04	1.54 ± 0.04
25FNSW	2378 ± 3	59 ± 1	4.11 ± 0.04	9.8 ± 0.1	33.6 ± 0.3	0.123 ± 0.03	1.53 ± 0.03

## Data Availability

Data is contained within the article.
